# Study on the experiment and reaction kinetics of sulfur removal from coal by microorganisms

**DOI:** 10.3389/fmicb.2023.1184253

**Published:** 2023-06-05

**Authors:** Dan Zhao, Ping-ping Sun, Chun-ming Ai, Xiao-zhi Mu

**Affiliations:** ^1^College of Safety Science and Engineering, Liaoning Technical University, Huludao, China; ^2^Key Laboratory of Thermal Disaster and Prevention, Ministry of Education, Huludao, China; ^3^Key Laboratory of Mine Thermodynamic Disasters and Control of Ministry of Education, Huludao, China; ^4^Shanxi Jinshen Shaping Coal Industry Co., Ltd., Xinzhou, Shanxi, China

**Keywords:** microbial desulfurization, coal spontaneous combustion, pyrite, reaction kinetics, activation energy

## Abstract

To solve the safety problem of spontaneous combustion of high-sulfur coal, applied microbiology, physical chemistry, reaction kinetics theory, combined with the SEM, FTIR and TG-DTG-DSC experiments and analysis of testing methods, the microbial desulfurization experiments were carried out, and the change law of the desulfurization reaction of coal before and after the element composition, main physical and chemical properties, the coal spontaneous combustion point was studied. The results show that when the temperature is 30°C, the coal particle size is 120 mesh, the initial pH value is 2.0 and the bacteria liquid amount is 15 mL, the desulfurization effect of the coal sample is the best, and the maximum desulfurization rate can reach 75.12%. There is obvious erosion on the surface of the coal sample after microbial desulfurization, the pyrite in the coal is obviously reduced, and the molecular structure in the coal is basically unchanged. Under the action of microorganism, part of inorganic sulfur in coal is removed, the spontaneous combustion point of coal is increased by 50°C, the activation energy of coal has increased more than three times, and the possibility of spontaneous combustion of coal is reduced. By analyzing the reaction kinetics of the microbial desulfurization process, it can be seen that the microbial desulfurization reaction is controlled by external diffusion, internal diffusion and chemical reaction, among which internal diffusion is the main influencing factor.

## 1. Introduction

Coal is an important basic energy and chemical raw material in China. The situation of rich coal, poor oil and little gas determines that China’s energy development must be based on coal, and coal will remain China’s main energy for a long time (Jie et al., 2021; Li, 2021). Coal spontaneous combustion is one of the most common disasters. Fire accidents not only cause casualties, equipment damage and resource waste, but also emit toxic and harmful gases to pollute the environment, causing incalculable losses (Liang et al., 2022; Qiao et al., 2022; Zhang et al., 2022).

Research by domestic and foreign scholars (Santos and Duarte, 2016; Xi and Shi, 2021; Zhang et al., 2021) have shown that, the sulfur content, mineral composition, spontaneous combustion point and activation energy of coal are all important factors that determine the process of coal spontaneous combustion. However, the pyrite in coal has a faster oxidation rate and has a strong self-ignition potential. Coal with high pyrite content is more prone to spontaneous combustion. Therefore, it is urgent to find a method to reduce the content of pyrite in coal.

At present, coal desulfurization methods mainly include physical method (Cano et al., 2018; Ait-Khouia et al., 2021), chemical method (Rehman et al., 2018; Xia, 2018; Ge et al., 2021; Xu et al., 2021), and biological method (Liu et al., 2019; Kotelnikov et al., 2020; Tang et al., 2021). Among them, microbial method has become the research focus and development trend in the field of coal desulfurization because of its clean and environmental protection, low process cost, simple process, low energy consumption, mild conditions and other characteristics. Rudolfs (1922) and Rudolfs and Helbronner (1922) firstly reported the bacterial leaching of pyrite and zinc sulfide ore. Colmer and Hinkle (1947) firstly isolated *Thiobacter ferrooxidans*, which can oxidize metal sulfide, from acid mine water in coal mines. Yang et al. (2015) used self-screened *Thiobacillus ferrooxidans* to react with coal, and finally the total sulfur removal rate reached 70%, including 80% pyrite removal rate. Liu et al. (2019) used the *Acidithiobacillus ferrooxidans* to conduct coal bio-desulfurization experiments. The results showed that the removal rates of pyritic sulfur and total sulfur from coal effectively increased. Xu et al. (2020) used *Thiobacillus ferrooxidans*, *Escherichia coli*, and *Pseudomomas pitida* to conduct desulfurization experiments on Shanxi high-sulfur coal, and the results revealed that the dominant strain was *Pseudomomas pitida* which attained 58.23% of total sulfur removal during 10 days. Xu et al. (2022) used *Nocardia mangyaensis and Pseudomonas putida* to remove organic sulfur from coal, and the organic sulfur removal of these two bacterial strains on high-sulfur coal were 61.58 and 54.19%, respectively. Chen et al. (2021) used the mixed culture of *A. ferrooxidans* and *A. thiooxidans* to bioleach coal gangue in a column reactor, and the desulfurization rates of the pyrite and sulfate were 78.79 and 49.02%. The biological method can effectively remove sulfur from coal, but the mechanism of interaction between microorganisms and coal, reaction kinetics and the change law of physical and chemical properties of coal still need further study.

In this paper, the experiment of microbial desulfurization of high sulfur coal is carried out. Firstly, the optimum conditions of microbial desulfurization are obtained by orthogonal experiment. Secondly, X-ray diffraction (XRD) and Fourier transform infrared spectroscopy (FTIR) and other methods were used to study the changes of physical and chemical properties of coal before and after microbial desulfurization. Finally, the kinetics of microbial desulphurization was analyzed, and the factors restricting the process of microbial desulphurization were determined, which provided a theoretical basis for the prevention and control of coal spontaneous combustion by microbial desulfurization.

## 2. Experimental materials and methods

### 2.1. Experimental materials

#### 2.1.1. Experimental coal samples

Coal samples were taken from a coal mine in Datong, Shanxi Province, and spontaneous combustion accident occurred in the coal storage pile of the coal mine. The industrial analysis results of coal samples were shown in Table 1. The total sulfur content in the coal sample is 2.39%, which belongs to medium high sulfur coal, and there is a great risk of spontaneous combustion. The sulfur content of pyrite is 1.35%, accounting for 56.49% of total sulfur. After mechanical crushing, the coal sample is divided into different particle sizes according to the test requirements and stored in seal.

**Table 1:** Industrial analysis of coal samples.

Project	Total water (%)	Analysis water (%)	Ash content (%)	Fixed carbon
Content	5	0.8	20	65
Project	Volatile matter (%)	Total sulfur (%)	Pyrite sulfur (%)	Calorific value (MJ/kg)
Content	18	2.39	1.35	28

#### 2.1.2. Experimental microorganism

The pit water of the coal mine was collected as stock solution for screening bacteria. 10 mL of filtered pit water was put into a conical flask with 100 mL of 9 K liquid culture medium (as shown in Table 2). The conical flask was put into a shaking table at 150 r/min and 30°C for culture until the solution was reddish-brown. And repeat the culture steps for many times. To purify the bacteria in the original solution, the supernatant of the cultured solution was cultured in 9 K solid, and a single colony was selected in 9 K liquid medium for further culture. The supernatant was coated in 9 K solid medium, and a single strain was selected for microscopic examination. Repeat the purification steps for many times until the microorganism with consistent morphology can be observed under the microscope, which is the pure strain screened.

**Table 2:** Composition of 9 K medium.

Serial number	Name	Chemical formula	Content (g/L)
1	Ammonium sulfate	(NH_4_)_2_SO_4_	3.0
2	Potassium chloride	KCl	0.1
3	Dipotassium hydrogen phosphate	K_2_HPO_4_	0.5
4	Magnesium sulfate	MgSO_4_·7H_2_O	0.5
5	Calcium nitrate	Ca(NO_3_)_2_	0.01
6	Ferrous sulfate	FeSO_4_·7H_2_O	44.2

**Figure 1:**
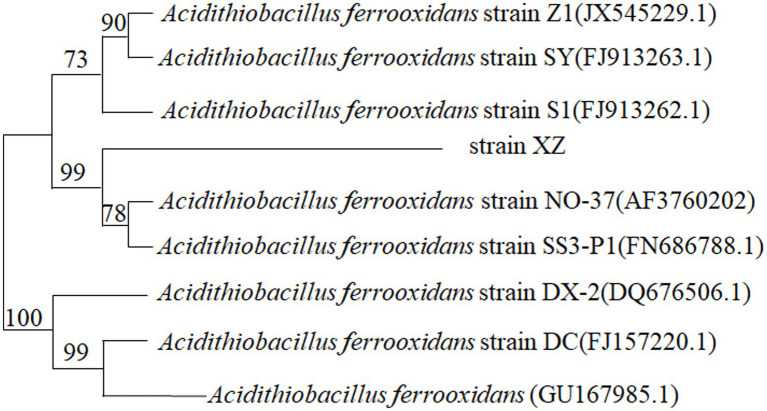
Phylogenetic tree of 16S rRNA sequence of strains.

In order to remove the precipitated impurities in the bacterial solution, the pure bacterial solution was placed in the centrifuge for 10 min, and the genome was extracted from the centrifuged bacterial body. 16S r RNA was amplified from the bacterial genome by PCR technology and then cloned and sequenced. The result was compared with the known sequence in GenBank to determine that the bacterial strain was *Aciditithiobacillus ferrooxidans* (*A. f*). The result is shown in Figure 1.

The main way for *A. f* to obtain energy for growth and metabolism is to oxidize Fe^2+^ in the solution to Fe^3+^. The higher the conversion rate of Fe^2+^ in the solution, the more energy *A. f* can obtain and the faster its growth and metabolism. Therefore, the conversion rate of Fe^2+^ in the solution can be used to characterize the growth activity of microorganisms. The conversion of Fe^2+^ in solution is calculated as shown in Equation 1. The concentration of Fe^2+^ in solution can be determined by potassium dichromate titration (Bernardez et al., 2021; Cheng et al., 2022).


(1) $$\eta =\frac{c_{0}-c}{c_{0}}\times 100$$

Where: *η* – Fe^2+^conversion rate in solution, %; *c*_0_ – initial Fe^2+^concentration in the system, g/L; *c* – instantaneous Fe^2+^concentration in the system, g/L.

### 2.2. Experimental principle

**Figure 2:**
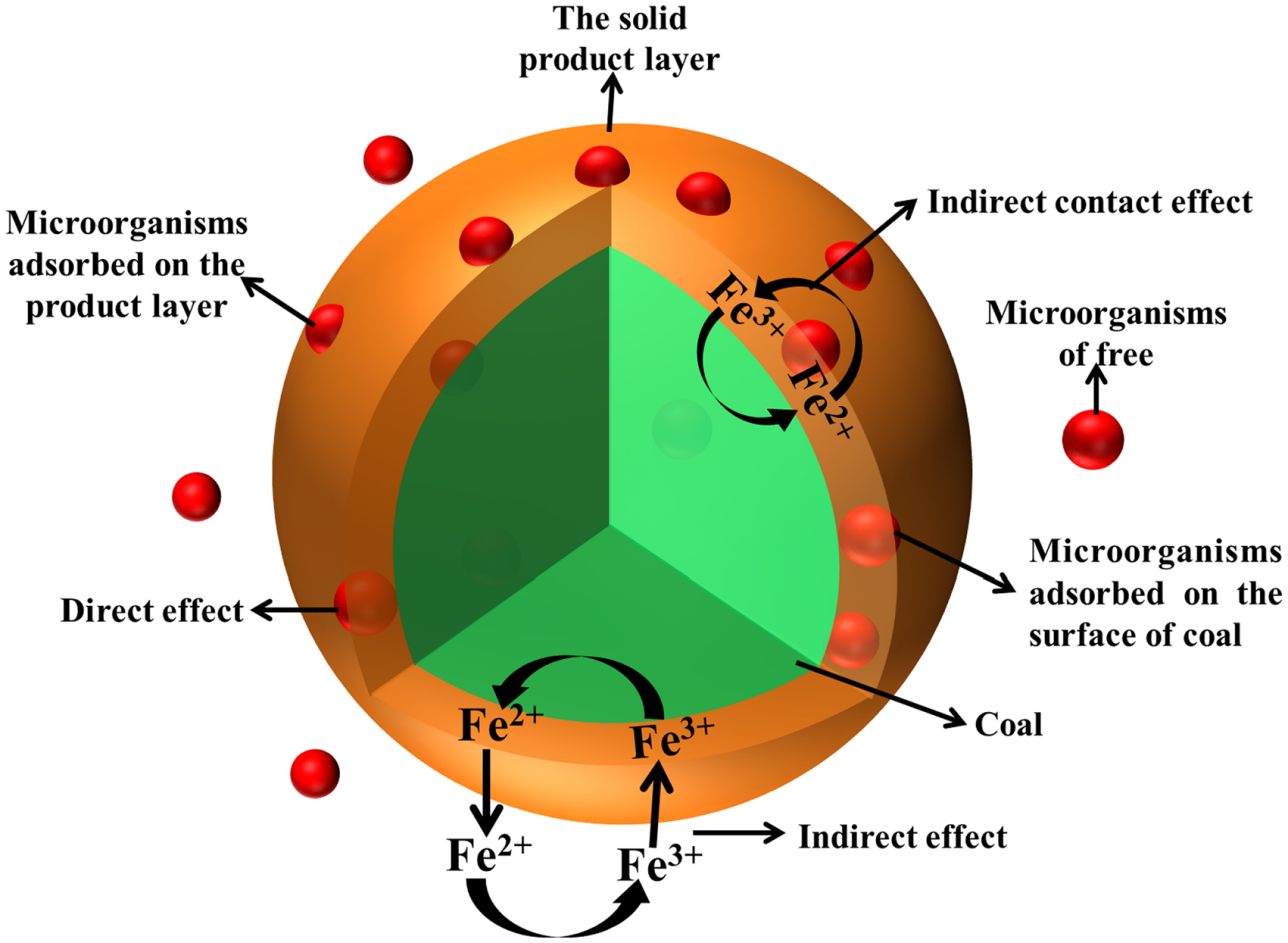
The mechanism diagram of microbial desulfurization process.

Microbial desulfurization process is a complex biological and chemical kinetic process. Microorganisms take place a series of redox reactions on the surface and inside of coal to oxidize sulfur on the surface and inside of coal. Microbial desulfurization is determined by both direct and indirect effects. The two effects are carried out at the same time, but sometimes the direct effect is dominant, sometimes the indirect effect is dominant (Pan et al., 2020; Xu et al., 2020; Ai et al., 2022). The principle of microbial desulfurization process is shown in Figure 2.

#### 2.2.1. Direct effect

The direct effect of microbial desulfurization is that microorganisms adsorb on the coal surface and directly oxidize and decompose the sulfur in the coal through protein secretion or other metabolites. The sulfur in the coal is oxidized and decomposed into Fe^2+^ and S (as shown in Equations 2, 3), and the microorganisms further oxidize S to H_2_SO_4_ (as shown in Equation 4).


(2) $$2{\mathrm{FeS}}_{2}+2H_{2}O+7O_{2}\overset{\mathrm{Microbial}}{\to }4H^{+}+4{\mathrm{SO}}_{4}^{2-}+2{\mathrm{Fe}}^{2+}$$

(3) $${\mathrm{FeS}}_{2}+8H_{2}O+7O_{2}\to 16H^{+}+2{\mathrm{SO}}_{4}^{2-}+15{\mathrm{Fe}}^{2+}$$

(4) $$2S+2H_{2}O+3O_{2}\overset{\mathrm{Microbial}}{\to }2H_{2}{\mathrm{SO}}_{4}$$

When there is a small amount of Fe^2+^ in the desulfurization system, the sulfur in the coal can only be dissolved by the oxidation of microorganisms, which is mainly the direct action of microorganisms.

#### 2.2.2. Indirect effect

The indirect effect of microbial desulfurization is that microorganisms exist in the desulfurization system in the solution and the sulfur in coal oxidation process of Fe^2+^ oxidized to Fe^3+^ (as shown in Equation 5). Fe^3+^ has strong oxidizability, which further oxidizes the sulfur in coal, and the sulfur in coal oxidizes to Fe^2+^ (as shown in Equations 6, 7), and Fe^2+^ is catalytically oxidized to produce Fe^3+^. Again and again, in the presence of oxygen, sulfur in coal is oxidized into sulfuric acid through different ways with the oxidation of sulfur in coal.


(5) $$4{\mathrm{Fe}}^{2+}+4H^{+}+O_{2}\overset{\mathrm{Microbial}}{\to }2H_{2}O+4{\mathrm{Fe}}^{3+}$$

(6) $${\mathrm{FeS}}_{2}+3{\mathrm{Fe}}^{3+}\to 3{\mathrm{Fe}}^{2+}+2S$$

(7) $$S+6{\mathrm{Fe}}^{3+}+4H_{2}O\to 8H^{+}+{\mathrm{SO}}_{4}^{2-}+6{\mathrm{Fe}}^{2+}$$

When there is a large amount of Fe^2+^ in desulphurization system, the process of free microorganisms obtaining energy by oxidizing Fe^2+^ is the indirect action of microorganisms. The microorganisms adsorbed on the product layer cannot obtain energy by oxidizing sulfur in coal, but can only oxidize Fe^2+^ in the system to survive, which is the indirect contact effect. The two processes together produce a large amount of Fe^3+^. Fe^3+^ enters the reaction zone of coal to oxidize sulfide through diffusion, and generates Fe^2+^ which diffuses into the solution and is oxidized. In such a cycle, sulfur in coal is gradually removed.

### 2.3. Experimental process and equipment

#### 2.3.1. Coal samples pretreatment process

**Figure 3:**
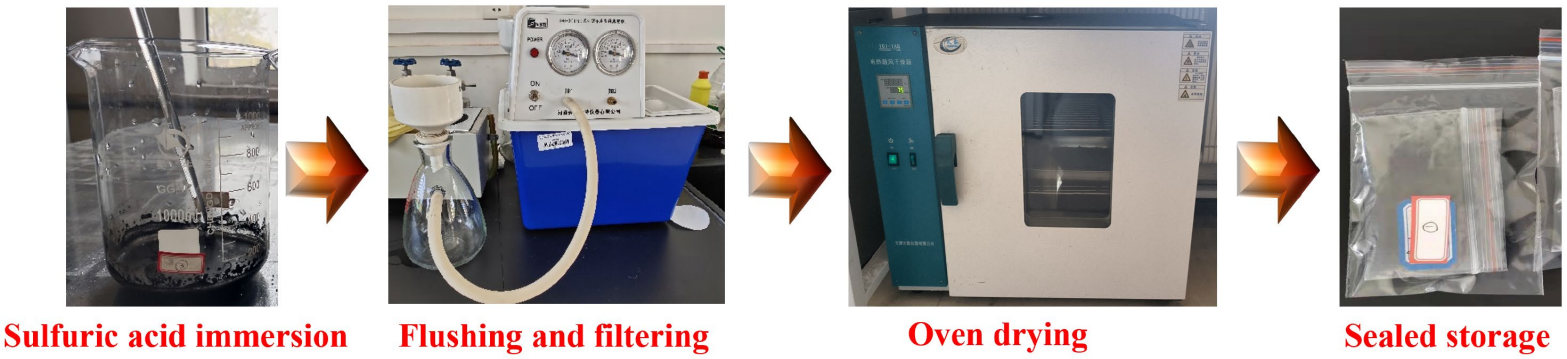
Flow chart of coal samples pretreatment steps.

To reduce the impact of soluble impurities in the coal sample on the microbial desulfurization effect, it is necessary to pretreat the coal sample (Chen et al., 2021; Rout et al., 2022), the steps were shown in Figure 3. Appropriate amount of coal sample was put into a beaker with sulfuric acid solution with pH = 1.5, soaked at room temperature for 24 h, filtered and washed with distilled water until the solution was neutral, dried and sealed for storage.

#### 2.3.2. Desulfurization experiment process

**Figure 4:**
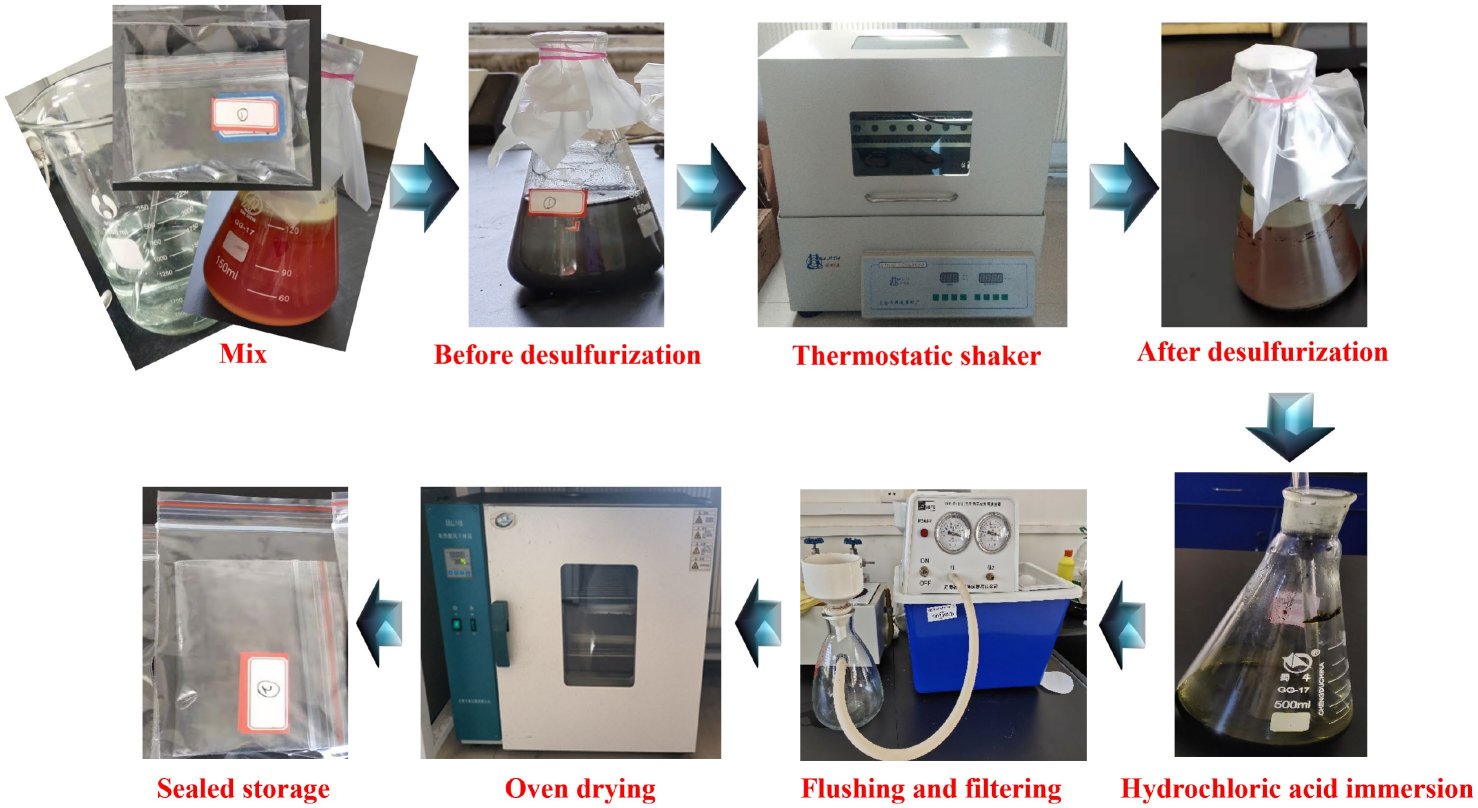
Flow chart of experiment process.

The desulfurization experiment processes were shown in Figure 4.

1. Experimental samples. According to the experimental scheme, the coal sample was broken and ground into different mesh numbers, and different amounts of coal sample and bacterial liquid were mixed with 9 K medium to prepare the experimental sample.
2. Desulfurization experiment. According to the experimental scheme, the prepared samples were placed in a shaker with different rotational speeds and temperatures for desulfurization experiments.
3. Sample processing. In order to dissolve the jarosite and other precipitates generated in the process of microbial desulfurization, the desulfurized samples were filtered, diluted hydrochloric acid was added to soak for 30 min, and distilled water was used to filter and wash until the solution was neutral.
4. Sample preservation. The processed samples were placed in the drying oven, dried at low temperature and then sealed for storage.

#### 2.3.3. Test for determination of physical and chemical properties of coal

**Figure 5:**
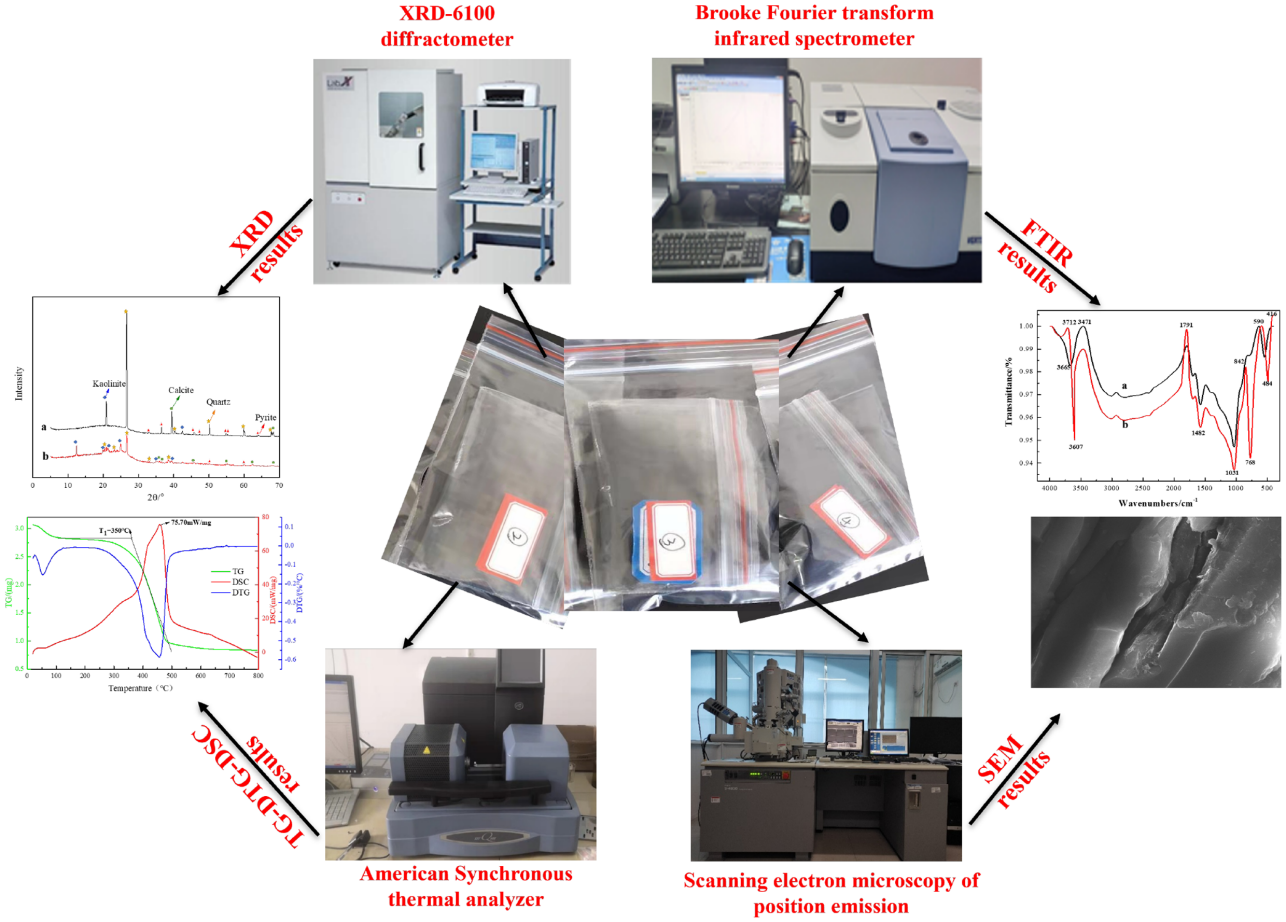
The experimental equipment and results.

In order to explore the influence of microorganisms on the physical and chemical properties of coal, the relevant physicochemical properties of coal before and after microbial desulfurization were tested. The experimental equipment and results are shown in Figure 5.

The functional groups and mineral components in coal were determined by Fourier transform infrared spectroscopy (FTIR) and X-ray diffraction (XRD), and the surface morphology of coals were detected by scanning electron microscopy (SEM) using Hitachi field emission scanning electron microscope. The thermogravimetric (TG-DTG-DSC) experiments were carried out using the American synchronous thermal analyzer to determine the spontaneous ignition point and activation energy of coals.

### 2.4. Experimental methods

In order to study the influence of various factors on the microbial desulfurization effect, the orthogonal experiment was used to investigate the four factors of temperature, coal particle size, initial pH value, and bacterial liquid quantity. Four levels were set for each factor without considering the interaction, as shown in Table 3. The desulphurization rate is taken as the investigation index, and the experimental scheme is shown in Table 4.

**Table 3:** Orthogonal experiment factors level table.

Level	Influencing factors			
Serial number	(A) Temperature/°C	(B) Coal particle size/mesh	(C) Initial pH value	(D) Bacterial liquid quantity/mL
1	20	60	1.5	5
2	25	80	2.0	10
3	30	100	2.5	15
4	35	120	3.0	20

**Table 4:** The experimental scheme.

Serial number	(A) Temperature/°C	(B) Coal particle size/mesh	(C) Initial pH value	(D) Bacterial liquid quantity/mL
1	1 (20)	1 (60)	1 (1.5)	1 (5)
2	1	2 (80)	2 (2.0)	2 (10)
3	1	3 (100)	3 (2.5)	3 (15)
4	1	4 (120)	4 (3.0)	4 (20)
5	2 (25)	2	3	4
6	2	1	4	3
7	2	4	1	2
8	2	3	2	1
9	3 (30)	3	4	2
10	3	4	3	1
11	3	1	2	4
12	3	2	1	3
13	4 (35)	4	2	3
14	4	3	1	4
15	4	2	4	1
16	4	1	3	2

Microorganisms (*A. f*) have little effect on organic sulfur, but it can remove most inorganic sulfur in coal. Therefore, this paper uses the amount of inorganic sulfur removed from coal as the desulfurization rate (as shown in Equation 8) to evaluate the desulfurization effect.


(8) $$w=\frac{\left(S_{B}-S_{A}\right)}{S_{B}}\times 100\%$$

Where: *w* – The desulfurization rate of coal sample, %; *S_B_* – the inorganic sulfur content in coal sample before desulfurization, %; *S_A_* – inorganic sulfur content in coal sample after desulfurization, %.

## 3. Experimental results and analysis

### 3.1. Analysis of experimental results of microbial desulfurization

#### 3.1.1. Range analysis of orthogonal experiment for microbial desulfurization

The range analysis of 16 groups of orthogonal experiments and their results was carried out, as shown in Table 5, from which it can be intuitively seen that the influence of various factors on the desulfurization rate of coal samples.

**Table 5:** Visual analysis of orthogonal experiment results.

Serial number	(A) Temperature/°C	(B) Coal particle size/mesh	(C) Initial pH value	(D) Bacterial liquid quantity/mL	The desulfurization rate/%
1	1	1	1	1	32.65
2	1	2	2	2	60.15
3	1	3	3	3	58.17
4	1	4	4	4	54.16
5	2	2	3	4	55.26
6	2	1	4	3	57.65
7	2	4	1	2	57.19
8	2	3	2	1	60.76
9	3	3	4	2	65.14
10	3	4	3	1	68.22
11	3	1	2	4	66.17
12	3	2	1	3	68.09
13	4	4	2	3	68.15
14	4	3	1	4	51.23
15	4	2	4	1	50.15
16	4	1	3	2	51.50
*K_1j_*	205.13	207.97	209.16	211.78	
*K_2j_*	230.86	233.65	255.23	233.98	
*K_3j_*	267.62	235.30	233.15	252.06	
*K_4j_*	221.03	247.72	227.10	223.82	
*^−^K_1j_*	51.28	51.99	52.29	52.95	
*^−^K_2j_*	57.72	58.41	63.81	58.50	
*^−^K_3j_*	66.91	58.83	58.29	63.02	
*^−^K_4j_*	55.26	61.93	56.78	56.71	
*R*	15.63	9.94	11.52	10.07	
Superior level	A_3_	B_4_	C_2_	D_3_	

*K_ij_* represents the sum of the experimental results at level *i* in column *j*, and *K_ij_* represents its average value.

In this experiment, the interaction between factors was not considered, and the desulfurization rate of coal samples for 10 days of the experiment was taken as the response value. Comprehensive analysis showed that the optimal experimental conditions for microbial desulfurization were A_3_B_4_C_2_D_3_, that is, the temperature was 30°C, the coal particle size was 120 mesh, the initial pH value was 2.0, and the bacterial liquid quantity was 15 mL. The experimental group was not included in the orthogonal experiment table, so the highest desulfurization rate was 75.12% under this condition.

#### 3.1.2. Variance analysis of orthogonal experiment for microbial desulfurization

The 16 groups of experiments and results of the orthogonal experiment were analyzed by variance, as shown in Table 6, from which the influence degree of each factor on the desulfurization rate of coal samples could be known.

**Table 6:** Analysis of variance in orthogonal experiment.

Source of variance	Degree of freedom	*Seq SS*	*Adj SS*	*Adj MS*	*F* value	*P* value
A	3	527.40	527.40	175.80	14.28	0.028^*^
B	3	208.84	208.84	69.61	5.65	0.094
C	3	365.97	365.97	121.93	9.90	0.041^*^
D	3	209.80	209.80	69.93	5.68	0.095
Error	3	36.95	36.95	12.32		
Total	15	1348.96				

*P* <0.01 is the extremely significant factor; ^*^ indicates significant factors; unmarked means insignificant factors.

In this experiment, L_16_(4^4^) orthogonal experiment was used, without considering the interaction, and the error of the four factors temperature (A), coal particle size (B), initial pH value (C), and bacterial liquid quantity (D) were 527.40, 208.84, 365.97, and 209.80, respectively. *p* value is an important index to test whether a factor has a significant impact on the experimental results. *p* < 0.01 is a very significant factor, *p* < 0.05 is a significant factor, and *p* > 0.05 is an insignificant factor. It can be seen from Table 6 that the order of significance of each factor on the experimental results was A > C > D > B, and temperature (A) and initial pH value (C) were significant factors.

#### 3.1.3. Theoretical analysis of significant factors of microbial desulfurization

1. Influence of temperature on microbial desulfurization

According to the Arrhenius Equation (Equation 9), the higher the temperature, the faster the microbial desulfurization reaction rate, the faster the microbial movement speed in the solution, the greater the convection and diffusion speed of bacterial solution, and the greater the desulfurization rate.


(9) $$k=A\cdot e^{-\frac{E}{RT}}$$

Where: *k* – reaction rate; *A* – frequency factor; *E* – apparent activation energy of reaction; *R* – ideal gas constant; *T* – absolute temperature.

However, the effect of temperature on the growth of microorganisms cannot be ignored. If the temperature is too high or too low, the microbial activity will be reduced and even death will occur. When the microorganisms were cultured at the optimum temperature, the growth and reproduction speed was the fastest and the activity was the strongest. At this time, the enzymatic reaction speed in the microorganism was the fastest and the desulfurization rate was high. Therefore, appropriate temperature is an important factor to determine the effect of microbial desulfurization.

1. Influence of initial pH value on microbial desulfurization

**Figure 6:**
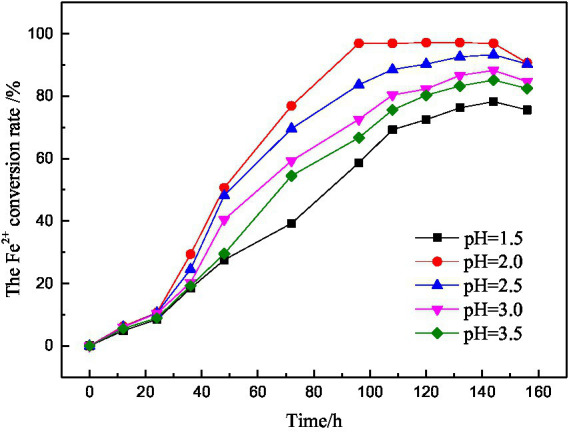
Fe^2+^ conversion at different initial pH values.

The initial pH value is an important factor that affects the growth and reproduction speed and activity of microorganisms. The growth of microorganisms with different initial pH values was shown in Figure 6. It can be seen that the growth trend of microorganisms with different initial pH values was roughly the same. The initial pH = 2.0–3.5, the microbial activity was from strong to weak, and the activity was the worst when the initial pH = 1.5. Therefore, it is known that the low or high initial pH value of the medium will inhibit the growth of microorganisms and further affect the microbial activity.

It is known from the experimental principle that the biochemical reaction in microbial desulfurization is an acid consumption process, which leads to the increase of pH value and the formation of solid products attach to the surface of coal particles, hindering the contact between microorganisms and coal particles and reducing the desulfurization rate.

### 3.2. Changes in physical and chemical properties of coal samples

#### 3.2.1. Change of chemical composition of coal samples

**Figure 7:**
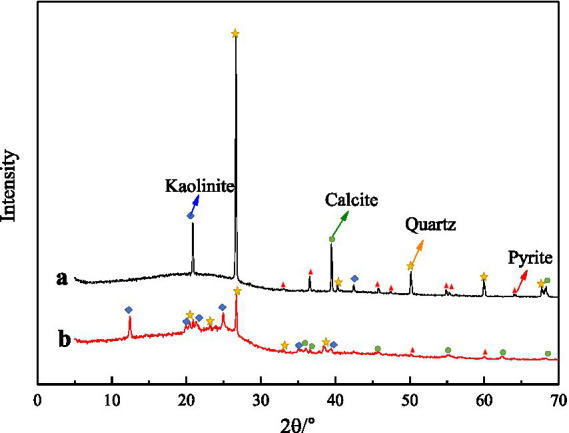
XRD spectrum of coal samples.

The main minerals in the coal samples used in this experiment are pyrite, calcite, quartz and kaolinite, etc. In order to understand the influence of microbial desulfurization on the properties of coal samples, the coal samples before and after desulfurization were taken for XRD analysis. The results are shown in Figure 7 (*a* is before desulfurization and *b* is after desulfurization). With the biochemical reaction between microorganisms and coal samples, some soluble minerals on the surface of coal samples were dissolved, so the mineral peak in coal samples after desulfurization was less than that before desulfurization. It can be seen from the graphs A and B that the material peak decreased significantly after 30°, especially for pyrite, because of the oxidation–reduction reaction occurred between coal sample and microorganism, indicating that microorganism can effectively remove pyrite and other inorganic sulfur in coal sample.

#### 3.2.2. Change of functional group of coal samples

**Figure 8:**
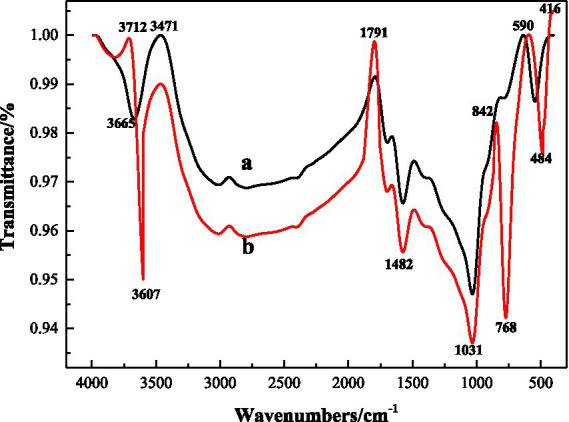
FTIR spectrum of coal samples.

According to Figure 8 FTIR spectra before and after microbial desulfurization (*a* is before desulfurization and *b* is after desulfurization), the shapes of FTIR spectra before and after microbial desulfurization are basically the same, indicating that there is no obvious change in organic functional groups in coal samples, and microorganisms mainly react with inorganic substances in coal samples. According to the comparison of the maps *a* and *b*, it weakens at the wave number of 3607 cm^−1^, which is due to the dissolution of kaolinite and other substances in the coal sample caused by microbial desulfurization and strong acid reaction environment. 1800 cm^−1^–1550 cm^−1^ is the C=O absorption band, and 1100 cm^−1^–1000 cm^−1^ is the S=O absorption band (Cai et al., 2021), and the intensity increases after microbial action. S-S vibration absorption is located at 590 cm^−1^, the absorption peak of pyrite at 416 cm^−1^, where the absorption intensity after desulfurization is obviously greater than that before desulfurization. After the action of microorganisms, some groups on the surface of coal samples have changed. The reason is that the protein molecules and polysaccharides and other substances secreted by microorganisms have physical and chemical reactions with coal, changing the surface properties of coal samples, so as to achieve the goal of desulfurization.

#### 3.2.3. Change of surface morphology of coal samples

**Figure 9:**
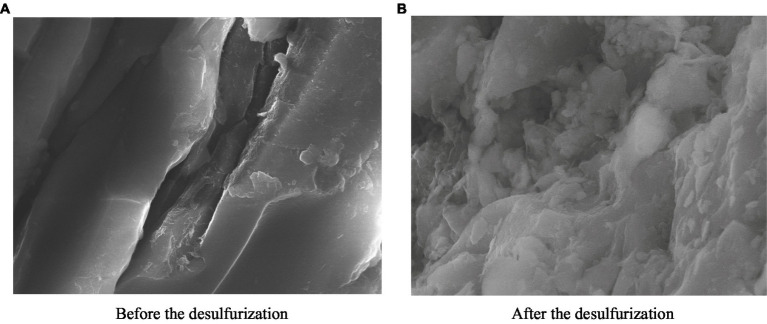
Surface morphology of coal samples before and after the test. **(A)** Before the desulfurization. **(B)** After the desulfurization.

The SEM images of coal samples before and after the microbial desulfurization experiment are shown in Figures 9A,B. In Figure 9A, the surface of the coal sample before the microbial desulfurization experiment is smooth and the structure is dense. After the desulfurization experiment, the micro morphology of the coal sample surface changed significantly (Figure 9B). The dense structure of the coal sample surface was dissolved and destroyed, becoming uneven and appearing corrosion morphology. The presence of pits on the surface of coal samples with the similar size as microorganisms strongly indicates that there is a reaction between microorganisms and the surface of coal samples.

#### 3.2.4. Change of ignition point of coal samples

**Figure 10:**
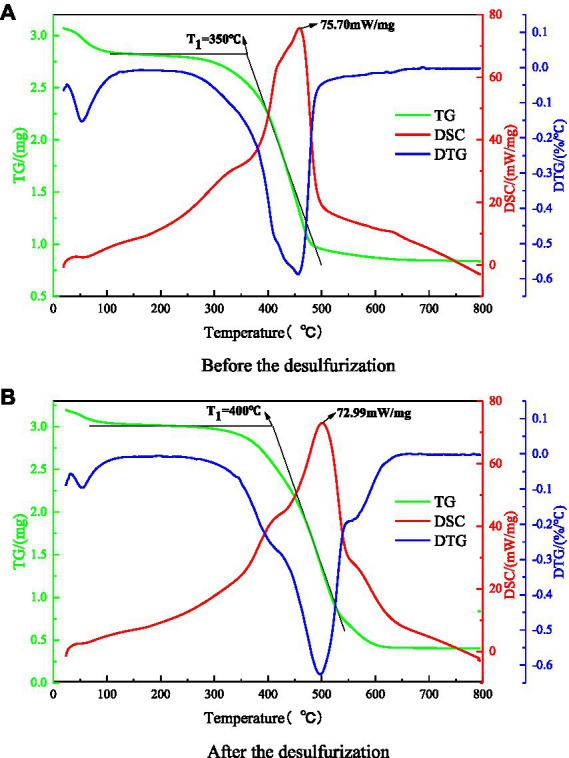
TG-DTG-DSC curves of coal samples before and after the test. **(A)** Before the desulfurization. **(B)** After the desulfurization.

The thermal analysis experiment was carried out by American synchronous thermal analyzer to explore the influence law of microorganism on coal thermal effect. Thermogravimetric experiments were carried out on coal samples before and after microbial desulfurization, and the TG-DTG-DSC curves of coal samples were obtained after smoothing treatment, as shown in Figures 10A,B. The characteristic temperature points of high adsorption temperature, critical temperature, dry cracking temperature, ignition point temperature, extreme temperature of heat flow rate and other characteristic temperatures can be determined from the curve. The ignition point and calorific value of coal are important factors that determine the spontaneous combustion process of coal. Therefore, this paper mainly focuses on the spontaneous combustion temperature (T_1_) and the extreme value of heat flow of coal samples.

The ignition temperature of coal refers to the temperature at which coal starts to burn, and the heat generated by coal during oxidation and reduction reaction is the heat flow rate. It can be seen from Figures 10A,B that after microbial desulfurization, the ignition temperature of coal sample is increased from 350°C to 400°C, and the extreme value of heat flow rate of coal sample is decreased from 75.59 to 72.99 mW/mg. The reason is that the sulfur content in coal is reduced under the action of microorganisms. Only a small amount of sulfide reacts with water and oxygen, and the water loss is faster and the water loss temperature is increased. The heat released by the adsorption of coal and oxygen and the oxidation–reduction reaction of sulfide causes the coal temperature to rise continuously until the ignition point. Microbial desulphurization reduces the concentration of sulfide in coal sample. The coal reacts inadequately with oxygen, which reduces the heat released and increases the ignition point temperature of coal. Therefore, the method of microbial desulfurization can effectively inhibit coal spontaneous combustion.

#### 3.2.5. Change of activation energy of coal samples

The molecule has to cross the energy barrier to participate in the reaction, and the activation energy is the lowest energy barrier that the molecule needs to cross. The activation energy can indicate the difficulty of chemical reaction. According to Arrhenius equation, the chemical reaction rate equation of coal is obtained as follows.


(10) $$\frac{dx}{dT}=\frac{A}{\alpha }e^{-\frac{E}{RT}}\left(1-x\right)$$

Where: *x* – the conversion rate of heating pyrolysis of coal; *T* – the thermodynamic temperature; A – the preexponential factor; *α* – the set temperature growth rate value, which is 10 in this paper; *E* – the activation energy of coal; *R* – the universal constant of gas, the value is 8.314.

By integrating Equations 10, 11 can be obtained.


(11) $$\ln\left[-\ln\left(1-x\right)\right]=\ln\left(\frac{AE}{\alpha R}\right)-5.314-0.1278\frac{E}{T}$$

**Figure 11:**
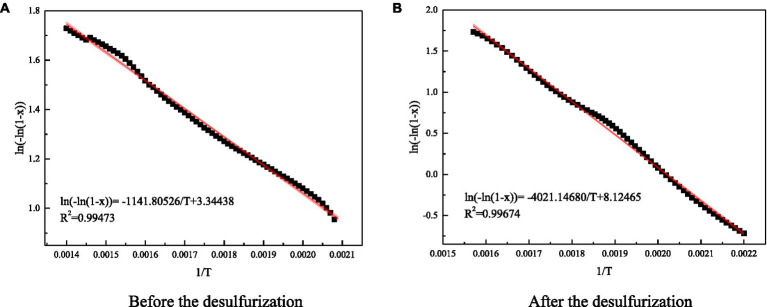
Fitting diagram of reaction dynamic coefficient of coal samples before and after the test. **(A)** Before the desulfurization. **(B)** After the desulfurization.

The curve of ln[−ln(1-x)] and 1/T was drawn, and then the linear equation of correlation of coal reaction kinetics was obtained by curve fitting. The value of activation energy could be obtained by using the slope of the curve, as shown in Figure 11.

The calculation shown that the activation energy of coal sample after microbial action increased from 8934.3 to 31464.4J/mol. Microorganisms removed the inorganic sulfur in coal, reduced the sulfide involved in the reaction, reduced the temperature inside and around the coal, slowed down the rate of coal self-heating, weakened the activation ability of active functional groups in coal, increased the reaction energy barrier of coal, coal samples are difficult to react. Therefore, microbial desulfurization can increase the activation energy of coal and delay the process of coal spontaneous combustion.

## 4. The analysis of reaction kinetics and desulfurization rate

**Figure 12:**
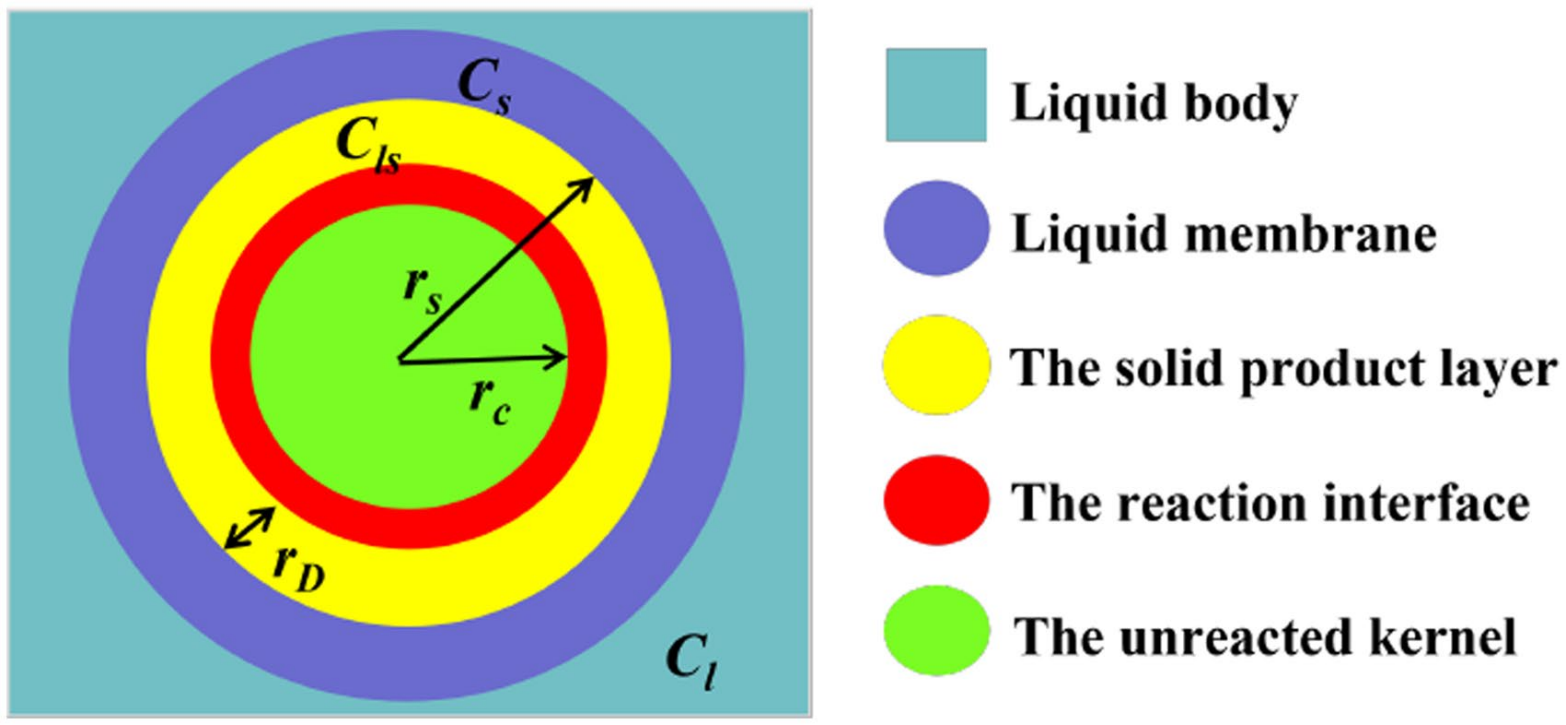
Desulfurization reaction diagram of coal particles.

At the same time, desulfurization rate is an important index to measure production efficiency and economic effect, which is mainly determined by microbial concentration and reaction kinetics in the process of desulfurization. The microbial desulphurization process conforms to the shrinkage model of solid–liquid interaction, which includes chemical reaction, external diffusion and internal diffusion control models. After contact with the coal, microorganisms reach the coal surface through the liquid membrane layer to react. At the same time, with the progress of desulfurization reaction, the chemical reaction products form a solid product layer on the surface of coal, as shown in Figure 12.

1. Microbial concentration change model

The *A. f* bacteria used in this article obtain growth energy by oxidizing Fe^2+^, and their maximum specific growth rate (*u_m_*) and yield coefficient (*Y*) are:


(12) $$\mathrm{Maximum specific growth rate:}\frac{dC_{l}}{dt}=\frac{\mu_{m}pC_{l}}{K+p}$$

(13) $$\mathrm{Yield coefficient:}C_{l}=C_{l}{}^{0}+Y\left(p^{0}-p\right)$$

After differentiation of Equation 13 and simultaneous Equation 12:


(14) $$\frac{dp}{dt}=-\frac{\mu_{m}p\left[C_{l}{}^{0}+Y\left(p^{0}-p\right)\right]}{Y\left(K+p\right)}$$

Where: *C_l_* is the microbial concentration in solution; *u_m_* is the maximum specific growth rate; *C_l_^0^* is the initial microbial concentration; *Y* is the yield coefficient; *p^0^* is the initial Fe^2+^ concentration in solution; *p* is the Fe^2+^ concentration in solution; *K* is the growth kinetic parameter.

By integrating Equation 14 and combining it with Equation 13, the model of microbial concentration change in the solution during microbial desulfurization can be obtained:


(15) t=[ln−ClCl0+KCl0Y+p0×ln−p0Cl0YClY(p0−ClY+Cl0Y)]/μm

By fitting and calculating the optimal culture conditions of microorganisms, *C_l_^0^*/*Y* = 1.0838, *K* = 0.328, *u_m_* = 0.0497. Change of microbial concentration in solution during microbial desulfurization can be obtained by substituting it into Equation 15.

1. Desulfurization reaction kinetics

The microbial desulfurization process is approximately steady-state, and in the presence of the solid product layer, the desulfurization process mainly includes:

1. Microorganisms reach the outer surface of coal through external diffusion in solution;
2. Microorganisms diffuse from the coal surface through the solid product layer shell to the surface of the unreacted kernel;
3. The unreacted kernel becomes smaller when microorganisms react with coal;
4. The reaction products leave the coal surface by diffusion.

The speed of microbial desulfurization is determined by both the chemical reaction at the interface and the mass transfer in the diffusion layer, which simultaneously affect the kinetics of the desulfurization process. For spherical geometry, the external diffusion rate, internal diffusion rate and chemical reaction rate are respectively:


(16) $$\mathrm{The external diffusion rate:}\frac{dn}{dt}=-\frac{4\pi r_{s}{}^{2}D_{1}\left(C_{l}-C_{s}\right)}{\gamma \delta }$$

(17) $$\mathrm{The internal diffusion rate:}\frac{dn}{dt}=-\frac{4\pi r_{s}r_{c}D_{2}\left(C_{s}-C_{ls}\right)}{\gamma \left(r_{s}-r_{c}\right)}$$

(18) $$\mathrm{The chemical reaction rate:}\frac{dn}{dt}=-4\pi r_{c}{}^{2}kC_{ls}$$

Where: *n* is the number of moles of unreacted inorganic sulfur in any time *t*; *r_s_* is the radius of coal particle; *D_1_* is the diffusion coefficient in the solution; *C_l_* is the microbial concentration in the solution; *C_s_* is the microbial concentration in the liquid membrane; *γ* is the stoichiometric coefficient; *δ* is the thickness of the diffusion layer; *r_c_* is the radius of the unreacted kernel; *D_2_* is the diffusion coefficient of diffusion through the product layer; *C_ls_* is the microbial concentration in the solid product layer; *k* is the desulfurization reaction rate constant.

Under the steady-state condition, the reaction rates of the three control models are equal to the total rate of the desulfurization process. Ignoring the reverse reaction rate, Equations 16–18 are solved simultaneously to obtain:


(19) $$C_{ls}=-\frac{1}{4\pi r_{c}{}^{2}k}\frac{dn}{dt}$$

(20) $$C_{s}=\left[-\frac{\gamma \left(r_{s}-r_{c}\right)}{4\pi r_{s}r_{c}D_{2}}-\frac{1}{4\pi r_{c}{}^{2}k}\right]\frac{dn}{dt}$$

(21) $$\frac{dn}{dt}=-\frac{4\pi r_{s}D2{}_{1}C_{l}}{\gamma \delta \left[1+\frac{r_{s}\left(r_{s}-r_{c}\right)D_{1}}{\delta r_{c}D_{2}}+\frac{D_{1}r_{s}2}{\gamma \delta r_{c}{}^{2}k}\right]}$$

The coal particles in the desulfurization reaction are approximately spherical in geometry, so it can be seen that:


(22) rs=rc(1−x)13

(23) $$n=\frac{4\pi r_{s}{}^{3}\rho }{3M}=\frac{4\pi r_{c}{}^{3}\left(1-x\right)\rho }{3M}$$

(24) $$\frac{dn}{dt}=-\frac{4\pi r_{c}{}^{3}\left(1-x\right)\rho }{3M}\frac{dx}{dt}$$

Where: *M* is the molecular weight of the reactant in coal; *ρ* is coal density; *x* is the reaction fraction, which is the desulfurization rate.

Combined Equations 24, 21 can be obtained as follows:


(25) dxdt=−3MClρ{γδrsD1+γrc2[1−(1−x)13]D2(1−x)13+rsk(1−x)23}

By integrating Equation 25, the relationship between microbial desulfurization rate (*x*) and time (*t*) can be obtained:


(26) t=A1x+A2[1−23x−(1−x)23]+A3[1−(1−x)13]

Where: $A_{1}=\frac{\gamma \delta \rho r_{s}}{3MC_{l}D_{1}}$ ; $A_{2}=\frac{\gamma r_{c}{}^{2}\rho }{2MC_{l}D_{2}}$ ; $A_{3}=\frac{\rho r_{s}}{MC_{l}k}$ .It can be seen from Equation 26 that the three items on the right of the equation represent external diffusion, internal diffusion and chemical reaction respectively, and the microbial concentration in the solution (*C_l_*) affects every link of the desulfurization process. By combining with Equation 15, the reaction kinetics of *A. f* bacteria in the desulfurization process with Fe^2+^ as the limiting nutrient can be obtained.

According to the relationship between desulfurization rate and desulfurization time, regression analysis was conducted on *A_1_*, *A_2_*, and *A_3_* in Equation 26 to determine the key factors restricting microbial desulfurization reaction. The fitting results are shown in Table 7.

**Table 7:** Regression of characteristic parameters of reaction kinetics.

*A_1_*	*A_2_*	*A_3_*	The correlation coefficient between and *t*			Regression coefficient *R*
			*r*(*A_1_*)	*r*(*A_2_*)	*r*(*A_3_*)	
−2839.16	−8547.56	7625.26	0.9495	0.9857	0.9616	0.9885

It can be seen from *r*(*A_2_*) > *r*(*A_3_*) > *r*(*A_1_*) that the contribution of the internal diffusion to the microbial desulfurization rate is greater than the other two items, the external diffusion has the smallest impact on the microbial desulfurization, and internal diffusion becomes the main link that restricts the desulfurization process. The internal diffusion mainly includes two processes: the diffusion of microorganisms through the coal surface pores and solid products to the reaction interface and the entry of desulfurization products into the solution. In the process of desulfurization, with the progress of the reaction, some solid products (such as jarosite, etc.) will cover the surface of coal and hinder microorganisms from reaching the reaction interface. So internal diffusion is the main factor determining the overall desulfurization rate. External diffusion refers to the process of microbial solution diffusing to the surface of solid products through the liquid membrane. Because the coal particles are immersed in the microbial solution, the coal particles can fully contact with the solution, and the external diffusion effect is relatively strong, so the external diffusion has little influence on the desulfurization process.

**Figure 13:**
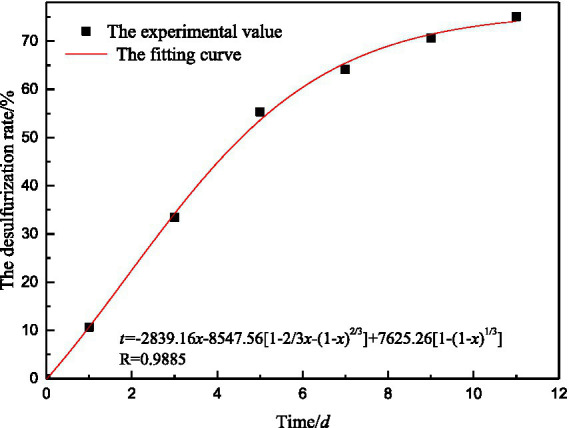
Fitting curve of coal particle desulfurization rate.

The fitting curve of desulfurization rate is obtained by combining the general equation of microbial desulfurization reaction kinetics and experimental results, as shown in Figure 13. The fitting curve of coal desulfurization rate is relatively close to the experimental value, and the regression effect is good.

## 5. Conclusion

1. The microbial desulfurization reaction conditions were determined. When the temperature is 30°C, the coal particle size is 120 mesh, the initial pH value is 2.0, and the amount of bacterial liquid is 15 mL, the desulfurization rate of coal sample reaches the highest.
2. Microbial desulfurization changes some functional groups in coal samples, but the change of macromolecular functional groups is small, which changes the surface properties of coal samples, and accelerates the process of microbial desulfurization reaction.
3. After microbial desulfurization, pyrite in the coal sample is significantly reduced, the corrosion pits appear on the surface of the coal samples, and the ignition point of the coal is increased by 50°C, the activation energy of coal samples increased significantly. The method of microbial desulfurization can effectively inhibit coal spontaneous combustion.
4. Through the analysis of reaction kinetics, it can be concluded that the microbial desulfurization reaction is controlled by external diffusion, internal diffusion and chemical reaction rate, among which internal diffusion has the greatest restriction on the reaction rate. Enhancing the internal diffusion of microbial desulfurization reaction can greatly improve the desulfurization rate.

## Data availability statement

The original contributions presented in the study are publicly available. This data has been deposited in the Genbank repository, accession number: QQ991324.

## Author contributions

DZ: methodology, investigation, and formal analysis. P-pS: data curation, software, and writing—original draft. C-mA: visualization, funding acquisition, and supervision. X-zM: visualization and investigation. All authors contributed to the article and approved the submitted version.

## Funding

This work was financially supported by the Education Commission of Liaoning Province (No. LJ2020JCL002) and the Natural Science Foundation Program of Liaoning Province (No. 2022-MS-395).

## Conflict of interest

X-zM was employed by Shanxi Jinshen Shaping Coal Industry Co., Ltd.

The remaining authors declare that the research was conducted in the absence of any commercial or financial relationships that could be construed as a potential conflict of interest.

## Publisher’s note

All claims expressed in this article are solely those of the authors and do not necessarily represent those of their affiliated organizations, or those of the publisher, the editors and the reviewers. Any product that may be evaluated in this article, or claim that may be made by its manufacturer, is not guaranteed or endorsed by the publisher.
